# FmocFF Peptide Hydrogel Is a Promising Matrix for Encapsulation and Controlled Release of the Anticancer Peptide Drug Bortezomib

**DOI:** 10.3390/biom15060839

**Published:** 2025-06-08

**Authors:** Peter Divanach, Antzela Noti, Panagiotis Vouvopoulos, Thanasis Athanasiou, Nikos Kountourakis, Vagelis Harmandaris, Anastassia N. Rissanou, Anna Mitraki

**Affiliations:** 1 Department of Materials Science and Engineering, University of Crete, Voutes Campus, GR-70013 Heraklion, Greece; petntiv@materials.uoc.gr (P.D.); angelanoti82@gmail.com (A.N.); bio1p502@edu.biology.uoc.gr (P.V.); 2 Institute of Electronic Structure and Laser, Foundation for Research and Technology-Hellas (FORTH), Nikolaou Plastira 100, Vassilika Vouton, GR-70013 Heraklion, Greece; athanasiou@iesl.forth.gr; 3 Proteomics Facility, Institute of Molecular Biology & Biotechnology, Foundation for Research and Technology-Hellas (FORTH), Nikolaou Plastira 100, GR-70013 Heraklion, Greece; kountour@imbb.forth.gr; 4 Institute of Applied and Computational Mathematics (IACM), Foundation for Research and Technology-Hellas (FORTH), GR-71110 Heraklion, Greece; harman@uoc.gr; 5 Department of Mathematics and Applied Mathematics, University of Crete, GR-71409 Heraklion, Greece; 6 Computation-Based Science and Technology Research Center, The Cyprus Institute, Nicosia 2121, Cyprus; 7 Theoretical & Physical Chemistry Institute, National Hellenic Research Foundation, 48 Vassileos Constantinou Avenue, GR-11635 Athens, Greece

**Keywords:** FmocFF Bortezomib, self-assembly, molecular dynamics, peptide hydrogels, controlled release

## Abstract

One major public health issue is cancer chemotherapy; despite constant progress in the area, administration of anticancer drugs to patients is often associated with serious side effects. It is therefore imperative to develop vehicles for encapsulation and controlled delivery of such drugs. Anticancer drugs include small peptide drugs, such as Bortezomib (BTZ). Self-assembling peptides have been recently reported as promising drug delivery agents. The research reported here proposes the encapsulation of BTZ into peptide hydrogels formed by the self-assembling FmocFF dipeptide as delivery vehicle. We selected FmocFF as an encapsulation vehicle based on our previous simulation study on the complexation propensity of Bortezomib (BTZ) with various peptide gelators. Herein we undertook additional computational studies that highlight the benefits of FmocFF as a potential effective nanocarrier for BTZ combined with experiments of encapsulation and evaluation of BTZ release. Based on these computational and experimental results, we propose the Fmoc-FF dipeptide hydrogel as a promising matrix for the controlled delivery of BTZ.

## 1. Introduction

Cancer chemotherapy is a major public health issue. Treatment of patients includes administration of a drug, and many times, a combination of drugs [1]. Anticancer drugs include small molecules, antibodies, proteins, and peptides that can be delivered orally, intravenously, or subcutaneously. Many anticancer drugs, despite their potency, cause severe side effects due to non-specific targeting and therefore require repeated injections to patients, risk of development of cellular resistance, etc. Development of delivery systems for controlled administration and target-specific delivery is therefore imperative for both improved therapeutic efficiency and improvement of the patient’s quality of life [2]. The development of nanocarriers for targeted delivery is especially timely and imperative [3]; on that front, recent research reported on the combination of theoretical and experimental approaches toward the design of peptide self-assembled nanocarriers that can encapsulate and deliver small-molecule cancer therapeutics [4,5].

However, limited reports exist for the development of self-assembling peptides as carriers for peptide anticancer drugs. Such a small anticancer drug peptide is N-Pyrazino-Phe-BoroLeu, called Bortezomib, hereafter referred to as BTZ. In 2003, BTZ was approved by the FDA for the treatment of refractory or relapsed multiple myeloma (MM), an incurable hematologic malignancy [6]. Its use was later extended for relapsed or refractory Mantle Cell Lymphoma (MCL) and other malignancies, including but not limited to non-Hodgkin’s lymphomas, solid tumors, and various cancer types, such as prostate, breast, ovarian, and more [7].

**Scheme 1:**
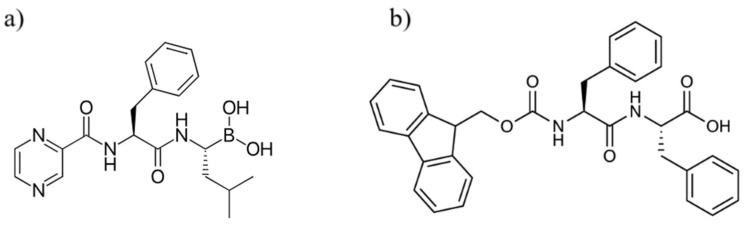
Chemical structures of (**a**) Bortezomib (BTZ) and (**b**) the FmocFF peptide.

Bortezomib (BTZ, *Velcade* ©), is a selective proteasome inhibitor that interferes with the ubiquitin-proteasome pathway [7], which is essential for the degradation of intracellular proteins. Chemically, BTZ is a boronic acid-dipeptide derivative comprised of a pyrazinoic acid blocked N-terminal end, L-phenylalanine, and L-leucine terminated in a boronic acid instead of a carboxylic acid (Scheme 1a). BTZ selectively targets the β5 subunit of the 20S proteasome, leading to reversible inhibition of proteasomal activity [8].

Despite its advantages, potency, and the reversible inhibition it offers, BTZ treatment is often still associated with several challenges. These include rapid clearance, causing the need for frequent intravenous or subcutaneous injections, severe side effects, and the development of resistance to the drug. Consequently, strategies involving the development of suitable carriers or scaffolds for controlled delivery of BTZ [9], including combination therapies [10] with BTZ [10,11] are areas of active research. Hydrogels might be such suitable carriers, offering a three-dimensional matrix with cross-linked functional properties and mimicking the natural extracellular matrix through exceptional water retention and adsorption [12]. These hydrophilic materials facilitate cell attachment, migration, and proliferation, making them suitable for biomedical applications such as tissue engineering [13] and drug delivery [14]. A thermo-sensitive hydrogel for BTZ drug delivery has been recently reported [15]. A peptide-based hydrogel, Nap-GFFYEEE-Cat (Nap standing for naphthalene and Cat standing for a catechol group), which chelates the boronate group of BTZ and releases it in acidic conditions, has been proposed for BTZ delivery [16]. Peptide-based hydrogels especially are biocompatible and biodegradable [17,18], with applications in drug delivery [19], wound healing [20], and tissue engineering [21]. Injectable hydrogels provide minimally invasive administration [22], reduce mechanical stress on cells, and allow targeted drug delivery [23] while reducing injection site reactions that can occur with subcutaneous administration of BTZ [24,25]. The FF dipeptide with the N-terminal (fluorenylmethyloxycarbonyl) protecting moiety (hereinafter referred to as FmocFF, see Scheme 1b) is a low-molecular-weight hydrogelator [26] forming hydrogels suitable for tissue engineering [27] and drug delivery [26]. FmocFF hydrogels can be formed with solvent-switch, pH switch, or catalytic methods (reviewed in [28]). The solvent-switch method is the most used and involves dissolution of the peptide powder in “good” solvent, such as HFIP, DMSO, or ethanol, followed by the addition of a “bad” solvent, mainly water, to trigger self-assembly [29]. FmocFF hydrogels can be combined with cationic amphiphilic peptides for delivery of cationic drugs [30] with dendrimers for delivery of indomethacin [31] or chitosan for delivery of the anticancer drug doxorubicin [32]. FmocFF hydrogels can also be formulated as nanogels for the targeted delivery of dexamethasone [33].

Based on the fact that most cyclic and protected dipeptides yield excellent hydrogels [27,29,34,35,36] with large surface areas, high water content, and adjustable pore sizes, in recent work, we screened and assessed the complexation propensity of certain aromatic and aromatic-aliphatic self-assembling dipeptides [37,38] with BTZ [39]. The evaluation of these dipeptides using a series of computational metrics indicated a strong complexation tendency between Fmoc protected diphenylalanine dipeptides and carboxybenzoxy-Phe-Phe and BTZ molecules. Comparing the two, as has been presented in ref. [39], the cluster formed by FmocFF peptides is looser (i.e., forms a less compact core compared to the one formed by carboxybenzoxy-protected FF). This can be attributed to the three-ring shape of the Fmoc protective group, which is of reduced flexibility in the way that it packs compared to the carboxybenzoxy-protection group. This is a feature that can potentially facilitate the release.

In the present manuscript, we opted to study FmocFF as the optimal candidate. We extended simulation runs of this specific system and performed additional analysis. Computational findings provide insights into molecular interactions, conformational properties, and the stability of FmocFF + BTZ complexes. We further characterized the rheological properties of the BTZ + FmocFF hydrogels, as well as their shear-thinning and recovery ability. Based on the theoretical predictions from the simulations that highlight the benefits of FmocFF as a potential effective nanocarrier for BTZ, we carried out the experimental test of encapsulation of BTZ inside FmocFF hydrogels, casting in tissue culture (TC) inserts and evaluating the release of the drug into the reservoir underneath the inserts. The release kinetics of BTZ from the FmocFF hydrogel matrix at 37 °C were evaluated with UV–Vis spectroscopy and mass spectrometry. The biological activity of the released BTZ was assessed through a Fluorometric Proteasome Activity Assay Kit utilizing a 7-amino-4-methylcoumarin (AMC)-tagged peptide substrate. Based on the theoretical and experimental results, we propose the FmocFF hydrogel as a a promising matrix for the controlled delivery of BTZ.

## 2. Materials and Methods

### 2.1. Model and Simulation

Simulations were conducted in aqueous solutions containing FmocFF dipeptides and BTZ molecules, similar to our previous work [39]. The systems were placed in a cubic simulation box and maintained under ambient conditions, with a temperature of 300 K and a pressure of 1 atm. Atomistic molecular dynamics simulations were carried out using the GROMACS software (GROMACS 5.1.4) [40], with explicit solvent representation based on the SPC/E water model [41]. Molecular structures were parameterized using the GROMOS54a7 force field [42], with force field parameters generated through the Automated Topology Builder (ATB) [43], a tool designed for molecular and complex system parameterization. Non-bonded interactions were described using a spherically truncated 6–12 Lennard–Jones potential, applying standard Lorentz–Berthelot mixing rules with a 1 nm cut-off. Electrostatic interactions were computed using the particle-mesh Ewald (PME) method, with a cut-off of 1 nm, a PME order of 4, and a Fourier spacing of 0.12. System pressure and temperature were maintained using the Berendsen barostat [44] and the stochastic velocity rescaling thermostat [45], with an integration time step of 1.0 fs. Post solvation, the initial configuration underwent energy minimization, followed by equilibration for at least 50 ns, before commencing production runs.

### 2.2. Experimental Details/Materials and Methods

#### Materials

The FmocFF peptide was obtained from Bachem (Bubendorf, Switzerland) in the form of lyophilized powder and had a purity greater than 99%. Ethanol solvent, when required, was provided by Sigma-Aldrich, and the water solvent used was nanopure purified, filtered, and sterilized. Acetonitrile (for MS analysis) was obtained from Fisher Scientific. The BTZ peptide was purchased from Merck (Darmstadt, Germany) in the form of lyophilized powder with purity greater than 95%. Ethanol and other chemical reagents were purchased from Sigma-Aldrich (St. Louis, MO, USA).

### 2.3. Methods

#### 2.3.1. BTZ Standard Curve

A total of 0.25 mg of BTZ was weighed in Eppendorf tubes and dissolved in 150 μL of EtOH (acting as the “good” solvent) added to each tube, then vortexed and sonicated to achieve the dissolution of the drug. A total of 350 μL of H_2_O (acting as the “bad” solvent) was added to achieve a solution with a 3:7 EtOH:H_2_O ratio for the good/bad solvent. Around 30 s of vortexing was applied to achieve appropriate mixing of the solution. The resulting BTZ sample had a final concentration of 0.5 mg/mL. UV–visible spectra were recorded in a UV–Vis spectrophotometer (UV-1700 PharmaSpec; Shimazdu Corporation, Kyoto, Japan) at a range of 360 to 220 nm, and spectra were subsequently recorded for serial dilutions. By evaluating the absorbance at 270 nm from the UV–Vis graph data, an average value was calculated for each set of data of different concentrations, and the standard curve was constructed (Figure S1).

#### 2.3.2. Gel Formation and Characterization

The FmocFF hydrogels were formed according to the solvent-switch method, where the FmocFF peptide powders were first dissolved into a “good solvent”, ethanol, while the “bad” solvent, water, was subsequently used to trigger self-assembly. Solutions were prepared by dissolving the FmocFF powder in EtOH, followed by sonication at 45 °C to facilitate dissolution. For the FmocFF + BTZ preparation, BTZ powder was added to the FmocFF solution in ethanol and sonicated for 1 min to achieve a mixture of the two peptides in ethanol. Water was subsequently added to the mixture to afford a final 3:7 EtOH/H_2_O ratio (Supplementary Videos S1 and S2 and Figure S2a,b). The control FmocFF sample without BTZ was also characterized in parallel with all methods. In order to evaluate the drug loading and encapsulation efficiency, three independent FmocFF + BTZ gels were prepared, and each gel was washed thrice with overlaying 1 mL of water each time. The absorbance spectra of the washes were recorded, the amount of non-encapsulated BTZ was calculated from the standard BTZ curve, and the total amount of non-encapsulated BTZ was calculated by adding the three values for each gel (total of nine). The weight of encapsulated BTZ was calculated by subtracting the amount of non-encapsulated weight from the initial BTZ weight. The drug loading and encapsulation efficiency are reported in Section 3.2.1.

#### 2.3.3. Rheological Characterization

Both FmocFF and FmocFF–BTZ hydrogels were characterized rheologically at 37 °C. Measurements were performed with an Anton Paar (Anton Paar GmbH, Graz, Austria) MCR501 shear rheometer equipped with a stainless-steel parallel plate geometry of 25 mm diameter. Evaporation was mitigated by an ethanol–water vapor saturated solvent trap. All experiments were performed in the strain control mode.

#### 2.3.4. Casting Fmoc-FF–BTZ Hydrogels and Release Protocol

The addition of ethanol and water to the FmocFF alone or the FmocFF + BTZ was followed by transferring the mixture to a 500 μL tissue culture (TC) insert with a 0.4 μm pore size (Sarstedt, Nümbrecht, Germany) and placement into a well. One mL of preincubated water at 37 °C was added to cover the bottom part of the TC insert. It is important to note that those samples gel in about 30 s; therefore, before complete gelation occurred, the resulting 500 μL of the sample were swiftly cast immediately after the addition of water via the micropipette. The resulting Fmoc-FF–BTZ samples had a concentration of the Fmoc-FF dipeptide at 2 mg/mL loaded with BTZ at a concentration of 0.5 mg/mL. In addition to the water added to the wells containing the samples, water was added to adjacent wells to prevent sample loss due to evaporation of the samples. The well plate had its covering placed on top of it and was transported inside an incubator adjusted to 37 °C for the entire experimental period. The experiment lasted for one week, and sampling was carried out during this period. The totality of water was retrieved and replaced again with preincubated water immediately after each sample retrieval.

A series of control FmocFF hydrogels were also prepared to evaluate the dissolution of the FmocFF matrix and detect the presence of dissolved Fmoc FF molecules in the release well. The same protocol as above was followed for casting the gels and collecting the samples. The sample collected immediately after casting and addition of the water was noted as sample t-0. The release evaluation was performed by using the following methods: UV–Visible spectroscopy, FESEM observations, and mass spectrometry analysis.

#### 2.3.5. UV–Visible Spectroscopy

The amount of BTZ released from the hydrogels in the water reservoir was quantified using UV–Vis spectrophotometry. UV–visible spectra were recorded at a range of 360 to 220 nm; BTZ has a maximum absorbance at 270 nm, reflecting the contribution of the phenyl and pyrazinoyl rings. Due to the Fmoc and phenyl rings, the FmocFF molecules also absorb at 270 nm; therefore, spectra were also recorded for the control experiment (FmocFF hydrogel only) to subtract the contribution of the FmocFF molecules released from the dissolution of the hydrogel over time (Figure S3).

#### 2.3.6. Field Emission-SEM (FESEM)

A total of 10 μL of the samples, collected at different time intervals, were deposited on glass slides and left to evaporate for 24 h at room temperature. After drying, the samples were placed on a slide using conductive carbon adhesive tape, and sputtering was performed for 64 s at 40 mA with 15 nm thick gold (Au) layer coating (Baltec SCD 050Bal-Tec AG, Pfäffikon, Switzerland ). The FESEM imaging was performed at the Biology Department of the University of Crete using a field-emission scanning electron microscope JEOL JSM-7000F operating at 15 kV and JEOL JFM-IT700HR (JEOL Ltd., Akishima, Japan) operating at 20 kV.

#### 2.3.7. Mass Spectrometry Analysis

For the mass spectrometry analysis, 20 μL of each FmocFF–BTZ release-solution in the water, collected at each time point, was mixed serially with 180 μL of acetonitrile spiked with 0.5% Formic acid. Extensive washes were performed between samples to avoid clogging and residual carry-over of BTZ between release-samples. Mass spectrometry analysis was conducted using an LTQ-Orbitrap XL-ETD (Thermo Scientific, Bremen, Germany) with an ESI max ion source (Thermo Scientific). Data acquisition was performed using Xcalibur-software version 2.1.0 (LTQ Tune 2.5.5 sp1, Thermo Scientific). Prior to analysis, the instrument was calibrated using a standard ESI positive ion calibration solution of caffeine (Sigma), l-methionyl-arginyol-phenylalanylalanine acetate H_2_O (MRFA, Research Plus, Barnegat, NJ, USA), and perfluoroalkyl triazine (Ultramark 1621, Alfa Aesar, Ward Hill, MA, USA). The mass spectrometer was operated with a spray voltage of 2300 V, capillary voltage of 35 V, tube lens voltage of 140 V, and a capillary temperature of 180 °C. Samples were injected at a flow rate of 3 μL/min, the mass range was 100–1500, and data were acquired with a resolution above 60,000 using the Orbitrap (Thermo Scientific, Bremen, Germany) analyzer.

#### 2.3.8. Proteasome Inhibitor Assay Protocol

Evaluation of the proteasome inhibitory activity was conducted using the proteasome fluorometric activity assay kit Abcam #AB107921 (Cambridge, UK). The assay is based on the release of a highly fluorescent 7-amino-4-methylcoumarin (AMC) from the AMC-tagged peptide substrate (Succ-LLVY-AMC) in the presence of proteasome-like activity. A Jurkat cell lysate rich in proteasome activity was included, as well as a specific proteasome inhibitor, MG-132. The assay was conducted according to the instructions of the manufacturer with opaque white well plates in a Synergy HTX BioTEK Plate Reader (BioTek, Bad Friedrichshall, Germany). The plate reader was preheated to 37 °C to ensure all reagents were maintained at the same temperature. The fluorescence of the released AMC was measured with excitation at 360 and emission at 440 nm.

## 3. Results and Discussion

### 3.1. Computational Evidence

Based on our previous work [39], FmocFF dipeptides were suggested as potential nanocarriers for BTZ. Validation of various dipeptides, based on a series of measures, indicated the strong complexation of protected diphenylalanine dipeptides (FmocFF and ZFF) with BTZ molecules. In the current work, additional simulation data are presented to assess whether the FmocFF hydrogel could be an effective delivery matrix for BTZ. The computational measures offer a complementary perspective to our experimental findings, shedding light on certain aspects of the molecular interactions, the conformational features, and the stability of the formed FmocFF–BTZ complexes. Initially, we calculated the potential of mean force (PMF), which is derived from constrained simulation runs involving only two molecules in solvent, one FmocFF and one of BTZ. In the following, the solvent accessible surface area (SASA) and the mean square displacement (MSD) were calculated in the aqueous solution of FmocFF and BTZ. The model system is described in Table 1.

#### 3.1.1. The Potential of Mean Force (PMF)

The effective interactions between dipeptides in water can usually be described by the potential of mean force (PMF), which is defined by the following Equation (1) [46]:

(1) $$U\left(Q\right)=-k_{B}Tlog\left(\int_{\Omega \left(Q\right)}e^{-U\left(q\right)/k_{B}T}\;dq\right)$$

In this equation, $q=\left(q_{1},q_{2},\ldots ,q_{N}\right)$ represents the positions of all N atoms in the system, and $Q=\left(Q_{1},Q_{2},\ldots ,Q_{N}\right)$ are the coordinates of the centers of mass (CoM) of the molecules. The integral is taken over all atomistic configurations corresponding to a given CoM-coordinate Q, denoted as $\Omega \left(Q\right)$, while V and T represent the system volume and temperature, respectively.

A common approach to simplifying this complex calculation is to estimate the PMF by evaluating the force acting on each molecule. This is expressed as follows:

(2) $$F_{i}\left(Q\right)=-\frac{\partial U\left(Q\right)}{\partial Q_{i}},\;i=1,\ldots ,M$$

where $F_{i}\left(Q\right)$ is the force on molecule i due to its interactions with all other molecules of the system. Typically, the many-body PMF is approximated using a two-body pair potential, which can be obtained through constrained simulations of two molecules in a solvent. In these simulations, the distance between the centers of mass of the two molecules is kept fixed, and the PMF $V\left(r\right)$ is computed by integrating the mean force $F\left(r\right)$ required to maintain the two molecules at distance r:

(3) $$V\left(r\right)=\int_{r_{\max}}^{r}F\left(r\right)\;dr-2k_{B}Tlnr$$

Here, $r_{\max}$ is the distance beyond which the interaction potential $U\left(r\right)$ tends to zero. The second term in the PMF expression accounts for the entropy correction due to the constraint imposed between the center of mass—center of mass distance, accounting for molecular rotations of the centers of mass [47].

**Figure 1:**
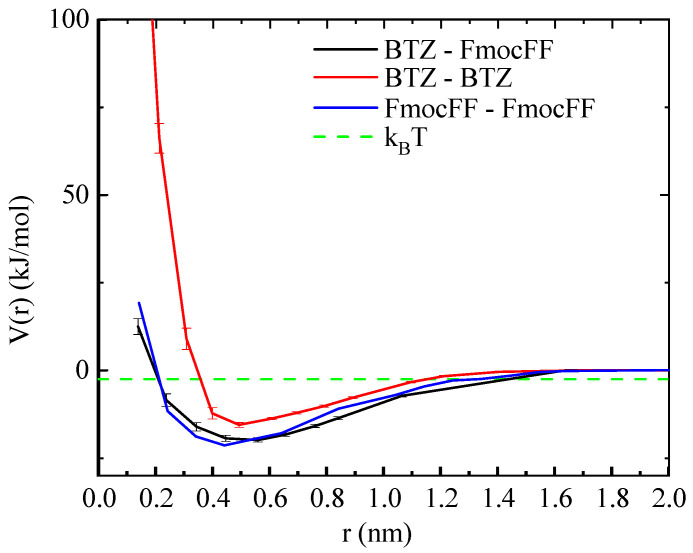
Potential of mean force (PMF) between BTZ–FmocFF (black), BTZ–BTZ (red), and FmocFF–FmocFF (blue) dipeptides at 300 K. The green horizontal line corresponds to the thermal energy *k*_B_*T*.

The calculation of PMF quantifies the propensity of the model dipeptides for self-assembly in aqueous environments. The PMF has been computed for all three pairs of molecules in water (i.e., FmocFF–FmocFF; FmocFF–BTZ; BTZ–BTZ), and the corresponding results are displayed in Figure 1.

**Figure 2:**
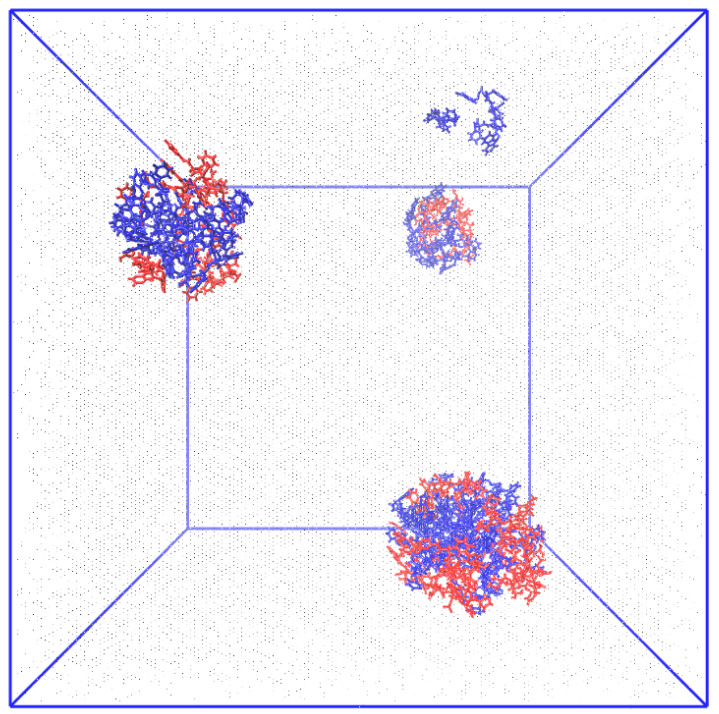
Characteristic snapshots from MD simulations of BTZ–FmocFF dipeptide complexes in aqueous solution. Water molecules are depicted as ghosts for clarity.

Notable attraction is observed in all three pairs, indicated by the attractive wells in the corresponding PMF curves. However, distinctions are evident between the BTZ–BTZ and the other two pairs (FmocFF–BTZ and FmocFF–FmocFF) with the last, exhibiting a deeper and wider attractive well. More specifically, the well is ranged between 0.2 and 1 nm with a minimum at around ~8.5 k_B_T for both FmocFF–BTZ and FmocFF–FmocFF, slightly shifted to longer distances for the former, while the BTZ–BTZ pair has a minimum at around ~6 k_B_T occurring between 0.3 and 0.8 nm. In all cases, at very short distances, repulsive interactions dominate, while beyond 1.6 nm, all effective interactions tend to zero. Therefore, the PMF data reveal a strong attraction between FmocFF and BTZ molecules, demonstrating a high assembly tendency. This interaction is comparable to the self-assembly propensity of FmocFF molecules and is much stronger than the self-assembly of BTZ molecules. These findings provide evidence that validates FmocFF as a viable scaffold for BTZ drug delivery. A characteristic snapshot of the complexation of the two molecules in water is presented in Figure 2.

#### 3.1.2. Solvent Accessible Surface Area (SASA)

The solvent accessible surface area (SASA) refers to the surface area of a molecule that is accessible to solvent molecules. The calculation of SASA involves representing the molecular surface as a series of points and then using a probe, typically a sphere, to determine the accessible regions. While several computational approaches are available today, one of the earliest and most straightforward methods is the Shrake–Rupley algorithm [48]. This technique distributes 92 points equidistantly around each atom and determines the accessible surface area by counting the number of points that are exposed to the solvent. The number of exposed atoms provides an estimate of the proportions of the total accessible surface area. GROMACS utilizes a variation of this approach designed to enhance computational efficiency known as the Double Cubic Lattice Method (DCLM) using the Eisenhaber et al. algorithm [49].

**Figure 3:**
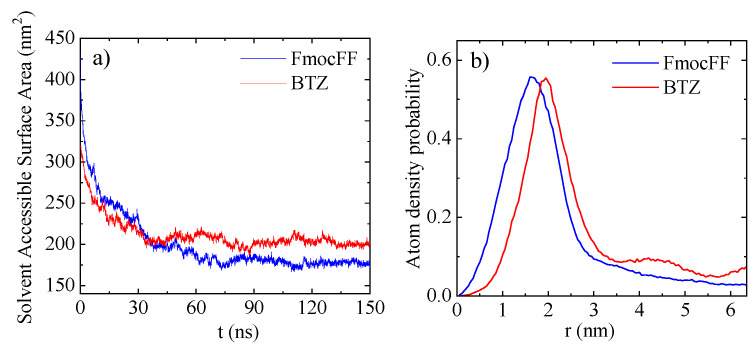
(**a**) The solvent accessible surface area (SASA) of BTZ and FmocFF molecules in the BTZ–FmocFF system in water at T = 300 K during the 150 ns run, and (**b**) Atom density probability of peptides and BTZ as a function of the distance from the center of the formed cluster, from our previous work [39].

The SASA was computed over the entire 150 ns duration of the simulation run for both the BTZ and FmocFF molecules in the aqueous solution and is presented in Figure 3a.

Initially high values of SASA are observed for both types of molecules since all molecules are uniformly distributed in the aqueous phase (Figure 3a). The slightly higher values of SASA for FmocFF compared to BTZ are attributed to the bigger size of the FmocFF molecule, which is quantified through the corresponding radius of gyration (i.e., R_g(_**_FmocFF_**_)_ = 0.513 ± 0.003 nm; and R_g(_**_BTZ_**_)_ = 0.425 ± 0.001 nm). A gradual decrease is observed in the following, when the clusters start to form, and, after ~50 ns, a steady-time-independent state is approached. Interestingly, at this state, the solvent accessible surface area is higher for BTZ molecules compared to the FmocFF ones. This observation indicates that BTZ is more “accessible” to water, and this lasts until the end of the simulation, although molecular rearrangements could still be observed between the clusters [39].

While this measure reflects the “packing” behavior during the clustering, it also provides another indication of BTZ likely being more “prone” and “accessible” to the water solvent, thus facilitating its release, compared to the FmocFF that is part of the hydrogel carrier. This behavior appears to be further reinforced by Figure 3b, where a detailed representation of the atomic arrangement within the cluster volume can be observed. The atom density probability of the different system components (BTZ and FmocFF) is thus provided as a function of distance from the cluster center. The concentration of BTZ molecules—and, consequently, boron atoms—is prominently higher at the cluster surface, rendering it more susceptible to water solvation.

#### 3.1.3. Mean Square Displacement (MSD)

The mean square displacement (MSD) is a metric for characterizing the diffusion of the particles and is based on the study of Brownian motion. MSD can be calculated following the Einstein relation [50]:

(4) $$\langle MSD\rangle =\underset{t\to \infty }{lim}\langle ||{\vec{r}}_{i}\left(t\right)-{\vec{r}}_{i}\left(t_{0}\right)|{|}^{2}\rangle =6Dt$$

where r is the position vector of a particle in three-dimensional space, i is the index of a particle in a system containing multiple particles, ${\vec{r}}_{i}\left(t\right),and{\vec{r}}_{i}\left(t_{0}\right)$ are the positions at time *t* and *t*_0_, and *D* is the translational diffusion coefficient.

MSD was computed for FmocFF dipeptides and BTZ molecules over the last 20 ns of the trajectory, where cluster formation has been completed and a “steady state” has been achieved.

**Figure 4:**
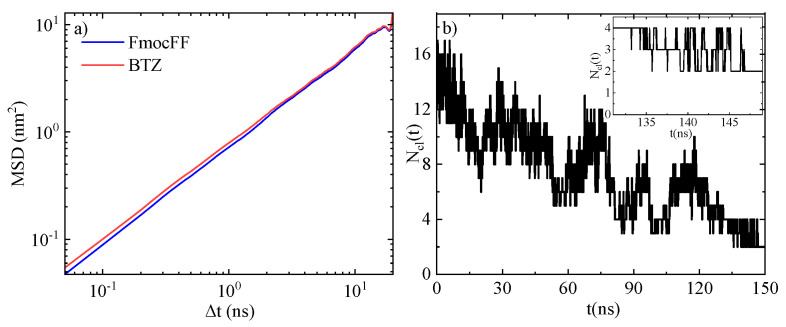
(**a**) The mean squared displacement (MSD) versus time of BTZ and FmocFF molecules in the BTZ–FmocFF system in water at T = 300 K, after the complex was formed and a “steady state was achieved” ($t_{0}=130\;$ns). (**b**) Number of clusters formed by BTZ and FmocFF as a function of time in the system; (Inset): Magnification on the last 20 ns of the trajectory.

The MSD plot of the BTZ and FmocFF in the BTZ–FmocFF complex system is presented in Figure 4a, suggesting a similar diffusion for both molecules. This displacement is measured in the post cluster formation period, showcasing the rather high mobility of both molecules within the complex, which may facilitate the drug release. It is important to note that this measure provides a rough estimation of the mobility of both molecules in a time window where a “steady” conformational state has been achieved. However, this state does not correspond to one completely stable cluster; thermal fluctuations exist, which lead to spontaneous attachment and detachment of individual molecules (either FmocFF or BTZ) from one cluster to another or even to merging or splitting between clusters. These events also contribute to the calculation of the MSD, overestimating its value. The evolution of the system towards a “steady” state is shown in Figure 4b, where the number of clusters formed by BTZ and FmocFF is presented as a function of time. A gradual decrease is observed as the clustering process takes place, including strong fluctuations, whereas at the last part of the trajectory, the system is almost stabilized, fluctuating between two and four clusters (Figure 4b inset). Therefore, our intention here is not to measure the rate of diffusion—a process influenced by cluster size and expected to be slower in larger aggregates due to energetic factors. Rather, we aim to provide a qualitative understanding of the substantial motion of dipeptide and drug molecules within the cluster and to discern variations in their mobility.

### 3.2. Experimental Results

#### 3.2.1. Gel Formation Kinetics, Drug Loading, and Encapsulation Efficiency

For the control FmocFF sample, immediately after the addition of water to the FmcoFF dissolved in EtOH, the mixture turned opaque and subsequently limpid, as known for the solvent-switch methods reported in [51] for the generation of FmocFF hydrogels. The optical transition reflects an initial nucleation phase into irregular aggregates that cause turbidity followed by subsequent rearrangements that lead to a limpid solution formed from fibrils [51]. In the 3:7 ethanol/water system used in the present study, the transition kinetics were very fast, and the mixture turned limpid in about 20 s (SI Videos S1 and S2). For the FmocFF + BTZ sample, the transition from opaque to limpid took about 25 s (SI Video S2). After 2 min for Fmocc FF and 2 min and 14 s for FmocFF + BTZ, the Eppendorf tubes were inverted, and self-supporting gels were observed for both (Figure S2a,b). The drug loading percentage was calculated as follows: (weight of encapsulated BTZ/weight of encapsulated BTZ + weight of FmocFF) ×100, and it was found to be 18.26 ± 1.05%. The encapsulation efficiency was calculated as follows: (weight of encapsulated BTZ/weight of initial BTZ) × 100 and found to be 88.11 ± 2.52%). Moreover, we have included a bar graph (Figure S4) for clarity. Of note, the choice of initial BTZ concentration of 0.5 mg/mL encapsulated in the present study was based on the following: preliminary studies were carried out to assess the inhibitory potential of various BTZ concentrations (). At this concentration, complete inhibition was observed compared to the control proteasome inhibitor (MG 132) supplied by the manufacturer (see also results Section 3.2.7), and it also allowed accurate measurements of UV–Vis spectroscopy and mass spectrometry.

#### 3.2.2. Rheological Properties of FmocFF and FmocFF + BTZ Complexes

**Figure 5:**
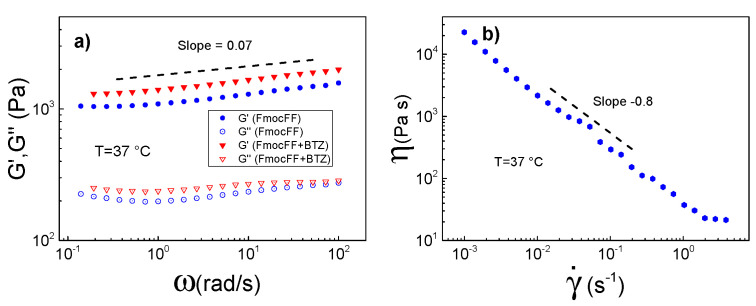
(**a**) Linear viscoelasticity of FmocFF and FmocFF + BTZ obtained by oscillatory shear at 0.2% strain amplitude; (**b**) steady shear response (viscosity) of FmocFF + BTZ as a function of shear rate. Dashed lines indicate the slopes of G′ (**a**) and η (**b**), respectively.

Linear viscoelasticity was characterized by small amplitude oscillatory shear tests. The storage modulus G′ and the loss modulus G″ of the complexes are depicted in Figure 5a. The weak power-law dependence of G′ on the angular frequency ω (slope of 0.07) is reminiscent of the self-similar network corresponding to a gel-like structure [52]. The encapsulation of BTZ does not weaken the gel response; on the contrary, it mildly enhances both moduli. Hence, in both cases, we have hydrogels with a modulus in the low kPa range and the same fractal structure (same slope). When exposed to steady shear flow with a gradually increasing shear rate, the FmocFF + BTZ gel exhibits a shear-thinning response, as shown in Figure 5b with a slope (thinning exponent) of 0.8, typical for hydrogels.

**Figure 6:**
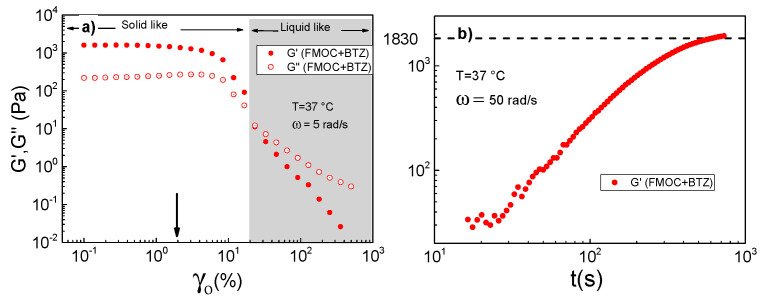
(**a**) Yielding behavior of FmocFF + BTZ during oscillatory strain amplitude (γ_ο_) sweep test. Non-shaded and gray-shaded areas mark the regimes with solid and liquid-like responses, respectively. The arrow indicates the linear limit of strain amplitude. (**b**) Recovery of FmocFF + BTZ network after yielding, probed with small-amplitude oscillatory shear. The dashed line indicates the value of G′ at 50 rad/s from Figure 5a. Time in the X axis is counted after the last point of the strain sweep in panel (**a**).

The yielding behavior was also studied with nonlinear oscillatory shear at large strain amplitudes and a fixed frequency of 5 rad/s (Figure 6). As the strain amplitude gradually increases, the response of the hydrogel departs from linearity at γ_ο_ > 0.2%. Initially, G′ gradually decreases while G″ exhibits a small increase. At 20% amplitude, the gel yields, as both moduli decrease significantly and G″ becomes greater than G′; i.e., it undergoes a solid to liquid transition, evidenced by the G′,G″ crossover. At the given frequency of 5 rad/s, the yielding shear rate amplitude is 1 s^−1^. This is the minimum rate needed to shear melt (liquify) the hydrogel. A gross estimate of the exhibited shear rate during a typical injection with a 21 G needle is two decades higher; i.e., 140 s^−1^ for the given needle [53]. This, along with the thinning of viscosity (Figure 5b), validates the injectability of this drug-encapsulating hydrogel.

The recovery of the gel network after yielding was probed by small amplitude oscillatory shear at 50 rad/s to minimize data acquisition time. Following yielding, depicted in Figure 5a, the storage modulus gradually recovered and attained values of 30 Pa at ≈ 20 s, while at 800 s, the gel’s network fully recovered, as shown in Figure 6b.

#### 3.2.3. Release Evaluation with UV–Visible Spectroscopy

The amount of BTZ released from the previously described FmocFF–BTZ hydrogel into water was collected at each respective time point and quantified using a UV–Vis spectrophotometer. As release medium, we used water to simulate the slightly acidic pH (around 6.5) surrounding the tumor microenvironment. Both BTZ encapsulated within FmocFF hydrogels and FmocFF hydrogel controls were run in parallel to dissociate the release of BTZ from the dissolution of FmocFF peptide molecules from the hydrogel matrix throughout the entire duration of the release experiment. Spectra were recorded from 360 nm to 220 nm, the maximum wavelength being 270 nm for BTZ. Samples were taken during 1 h, 3 h, 6 h, 12 h, 24 h, 48 h, 3 days, 4 days, 5 days, 6 days, and 7 days (Figure S5). For each time point, the absorbance due to the gradually released FmocFF alone was subtracted from the absorbance measured for the released BTZ. Release data were averaged from thirteen independent measurements at 37 °C. Cumulative BTZ release was calculated using the following equation:

$$Cumulative\;Release\left(\%\right)=\frac{Amount\;of\;drug\;released\;at\;time\;t+Amount\;of\;drug\;released\;at\;time\;t-1}{Initial\;amount\;of\;drug\;encapsulated\;in\;gel}\times 100$$

**Figure 7:**
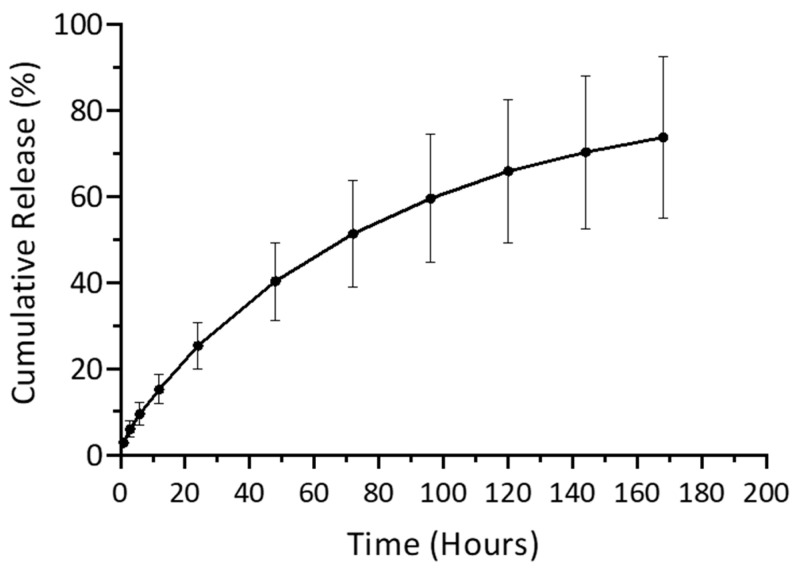
Graph depicting the cumulative release of BTZ from the Fmoc-FF hydrogel (mean + SD (n = 13)).

This formula calculates the percentage of drug released at a given time (t) by adding the amount released at time (t) and the amount released at the previous time point (t − 1), relative to the total initial amount of drug encapsulated within the hydrogel. The cumulative release curve is shown in Figure 7.

The time constant (τ) for the release of BTZ has been calculated to be 74 ± 12 h, corresponding to a complete release in 12 days.

#### 3.2.4. Structural Characterization with FESEM

Alongside the release experiments, samples were collected immediately following casting of the FmocFF–BTZ hydrogel into the TC inserts and after 48 h for structural characterization with FESEM imaging. We previously reported that BTZ itself self-assembles into spheres [39] (see also Figure S6a), whereas it is well established in the literature that the Fmoc-FF peptide self-assembles into a fibrillar network that forms hydrogels [29,39,54] (see also Figure S6b,c).

**Figure 8:**
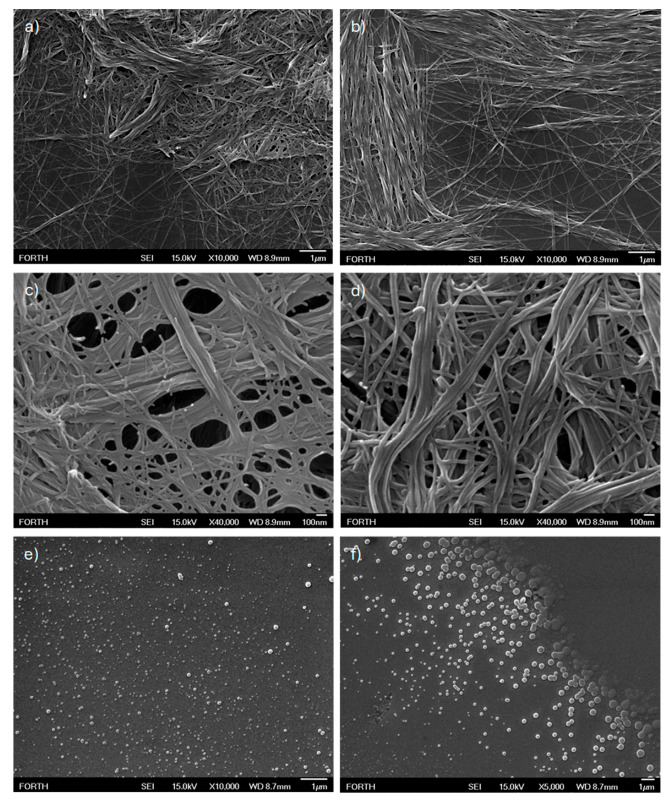
FESEM images of Fmoc-FF–BTZ samples: Images (**a**,**b**) correspond to a gel sample at 1:20 dilution ratio, retrieval time after initial gel deposition t = 0 s. Scale bars: 1 μm. Images (**c**,**d**) correspond to a gel sample at a 1:20 dilution ratio, retrieval time after initial gel deposition t = 48 h. Scale bars: 100 nm. Images (**e**,**f**) correspond to the retrieved undiluted eluate taken underneath the well plate, retrieval time after initial gel deposition t = 48 h. Scale bars: 1 μm.

In detail, upon placement of the sample in the TC insert, a piece from the formed hydrogel was retrieved and diluted inside an Eppendorf microtube at a ratio of 1:20 by the addition of water. Then, a 10 μL sample was retrieved for FESEM imaging; the abovementioned sample was considered as the t = 0 sample. Samples were retrieved following the same experimental protocol after 48 h. Additionally, 10 μL from the water reservoir surrounding the TC insert were retrieved at 48 h without being diluted to image the BTZ released at that time point. At both t = 0 and t = 48 h, fibrillar networks could be seen for the hydrogel samples, whereas the sample collected from the water reservoir at 48 h showed spheres. Figure 8 shows selected FESEM images obtained.

Co-assembly of fibrillar and spherical morphologies (for example, in the form of “beads-and-strings” [55]) was not observed; only fibrillar morphologies were observed, and the fibrillar networks at t = 0 and t = 48 h do not present significant morphological differences. Release and self-assembly of BTZ into spheres after 48 h was observed. The release kinetics of both BTZ and FmocFF were subsequently followed with mass spectrometry.

#### 3.2.5. Release Evaluation with Mass Spectrometry

**Figure 9:**
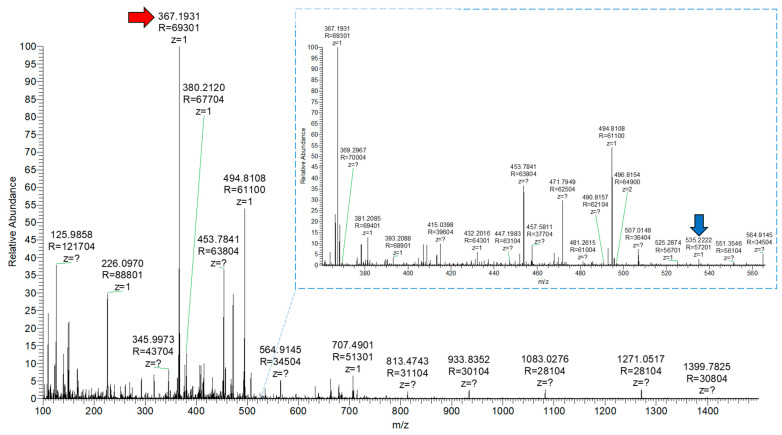
Product ion scan of BTZ (precursor *m*/*z* 367.19, red arrow) obtained in ESI positive ion mode for the 48 h FmocFF + BTZ release-sample in water at 37 °C; the minor peak at *m*/*z* 535.22 (blue arrow) corresponds to FmocFF.

As mentioned previously, the BTZ (exact mass: 384.2) peaks from FmocFF–BTZ release-samples were detected in positive ion mode with the help of an ESI source and the Orbitrap analyzer. The BTZ ions were labile and underwent in-source dehydration to form [M + H-H_2_O]^+^, causing the following BTZ-attributed peaks to be observed, within a 5 ppm tolerance, at *m*/*z* 226.09, 366.19, 367.19 (precursor *m*/*z*, Figure 9), and 368.19, and further validated by the MoNA–MassBank of North America [56].

Minimal BTZ relative intensities were observed for the early 1 h, 3 h, and 6 h samples. The peaks’ relative intensities reached a maximum value for the 48 h sample and the 3-day sample, followed by the 24 h and 4-day samples (see Supporting Information Figures S7–S17). Overall, the mass spectrometry results suggest that the relative intensities of released BTZ are minimal until 6 h, start to rise from 12 h onwards, and reach a maximum value at 48 h and 3 days. The drug continues to be released from 4 days to 7 days, albeit with lower intensities than the 48 h sample. Another much smaller peak intensity-wise, at *m*/*z* 535.22, could also be observed, which was attributed to the FmocFF ions. This peak became slightly more prominent (with a maximum relative intensity of up to and around 5–14%, depending on replicates, while the BTZ precursor peak reached 100) as the hydrogel further dissolved from 48 h onwards. It is important to note that FmocFF relative intensity peaks always remained very low, even for the 48 h and 3-day samples, where they reached their maximum values. Their normal relative intensity peak values were not detectable for the early 1–12 h samples and ranged between 1 and 3% for the one-week samples. Overall, the comparison of the BTZ and FmocFF release results suggests a clear-cut difference of release timing between them. The BTZ peak is detected from 6 h onwards without burst release observed, and reaches a maximum value at 48 h. On the contrary, the FmocFF peak becomes detectable at 48 h and remains at minimal intensities for the one-week sample.

#### 3.2.6. Discussion of the Possible Drug Release Mechanism

Taken together, the combination of results from release kinetics, FESEM characterization, and release evaluation with mass spectrometry converge toward a gradual release model of BTZ peaking at 48 h, whereas FmocFF molecules started to be released from 48 h onwards, and in minimal amounts compared to BTZ. The complete release (12 days) precedes the complete degradation of the FmocFF hydrogel, previously reported to be 17 days [54]. Moreover, no burst release has been observed for BTZ, suggesting that BTZ may not be freely encapsulated within the pores of the gel and released by diffusion. Rather, simulation findings reveal a complexation between the two molecules (BTZ and FmocFF) throughout the volume of the formed clusters. These experimental results correlate with the simulation results that suggest a strong assembly propensity between the peptides and BTZ, leading to the formation of a combined cluster. Structural arrangements of the molecules within the cluster reflect packing characteristics, such as the positioning of the drug partly on the outer surface, which may potentially expedite its release. The relatively high mobility of individual molecules within the cluster is also an indication of facilitated drug release. Similar results were presented in ref. [5], where the co-assembly properties of Cyclo-Histidine-Histidine (Cyclo-HH) peptides with six cancer drugs were investigated. The higher probability of direct or indirect interactions between drug and peptide nanocarriers within the co-assemblies was correlated with significantly improved drug encapsulation. The co-assembled clusters were formed by mixed distributions of peptides, ions, and drugs, with each component variably oriented toward either the core or surface, driven more by interaction patterns than by fixed spatial positions. Moreover, the study showed a linear relationship between the association-free energy of drugs and their solvent exposure within the co-assembled clusters. Drugs more buried in the clusters exhibited lower association-free energy, indicating stronger binding to the nanocarrier. This suggests a corresponding effect on their release process.

#### 3.2.7. Evaluation of the Inhibitory Activity of Released BTZ

**Figure 10:**
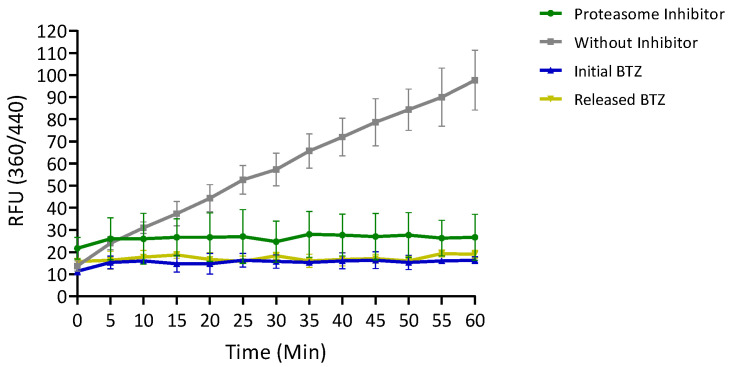
Kinetics of release of the fluorescent AMC product in the assay without inhibitor (gray curve), in the presence of MG-132 inhibitor (green curve), in the presence of the initially encapsulated BTZ (blue curve), and in the presence of BTZ released at 48 h (yellow curve). Mean ± SD (n = 3).

To evaluate the ability of released BTZ to inhibit the proteasome, four different solutions containing 1 μL of the substrate were prepared, each to a final volume of 100 μL. The first solution did not contain any proteasome inhibitor. The second solution contained 1 μL of the proteasome inhibitor MG-132. The third solution contained 1 μL of released BTZ at 48 h, and the fourth solution contained the starting BTZ concentration that was used for encapsulation in the FmocFF hydrogel. Subsequently, all prepared solutions were added to the preheated plate and incubated at 37 °C for 5 min to allow equilibration. Following this initial incubation, 2 mL of Jurkat cells were introduced into each well containing the prepared solutions to trigger the enzymatic reaction. The plate was subsequently placed in the plate reader, and measurements were recorded every 5 min over a 1 h period at 37 °C. The kinetics of release of the fluorescent AMC product are presented in Figure 10.

Though not quantitative, this assay confirms that the released BTZ maintains the ability to inhibit the proteasome activity following encapsulation and release from the FmocFF hydrogel matrix.

Overall, the simulation results presented above suggest a strong complexation propensity of BTZ with FmocFF, leading to clusters with conformational features that potentially favor BTZ release. The experimental results showed that the encapsulation of BTZ within the FmocFF hydrogel does not alter the hydrogel mechanical properties; the FmocFF + BTZ hydrogel presents shear-thinning and recovery properties, which validate its injectability. BTZ is released in a biologically active form following a cumulative release pattern without burst release. Its release precedes any dissolution of the gel matrix, which remains minimal during the time frame of one week, where the majority of BTZ is released (Figure 8 and Figures S7–S17). The timing for hydrogel dissolution is one of the major concerns when hydrogel carriers are used. The gel should disintegrate/dissolve much more slowly than the drug, as it was presented above, ensuring that the effects observed are due to the drug itself and not to the molecules of the carrier. Nevertheless, the effect of gel dissolution and the leaching of gelator molecules on both healthy and cancer cells must be considered. A few previous studies reported good viability of normal cell lines, such as chondrocytes and fibroblasts, in contact with, or following incorporation within, FmocFF gels [26,27]. FmocFF hydrogels with encapsulated small-molecule chemotherapeutics, such as 5-fluorouracil and Paclitaxel, were cast on TC inserts, and the effects of both the therapeutics themselves and the leachates from the gel were evaluated on model cancer cell lines [57]. The authors reported that apoptotic effects caused by the drugs on cancer cell lines could be distinguished by necrotic effects caused by the FmocFF molecule leachates, the latter occurring at times > 72 h. The concentration of FmocFF used in the study [57] was in the range of 1.5–3 mg/mL, i.e., close to 2 mg/mL used in the present study. As mentioned above, the relative intensity peaks of FmocFF molecules in mass spectrometry always remained very low, even for the 72 h samples, where they reached their maximum values, ranging between 1 and 3% for the one-week samples. The stability of the self-assembled peptide gelators toward dissolution and the slow release of their monomers in the surrounding environment is also an important parameter to consider when in vivo applications are envisioned. Nevertheless, it should be kept in mind that in vivo environments are different, and the exact mechanisms of both the drug release and dissolution of the gelator must be evaluated in the relevant context.

## 4. Conclusions

The development of nanocarrier systems for the delivery of cancer therapeutics is an active area of research with significant potential in treatment of the disease. Amongst the potential carriers, self-assembling peptides offer many advantages, such as biocompatibility, biodegradability, and designability at the sequence level. Anticancer peptide drugs have been less investigated compared to small-molecule anticancer drugs. In this manuscript, we combined theoretical and experimental approaches toward encapsulation and controlled release of a peptide anticancer drug, BTZ, by a self-assembling peptide hydrogel, formed by the peptide FmocFF as potential carrier. The integrated computational and experimental approach presented above consolidated the rationale for choosing FmocFF as a viable hydrogel scaffold for BTZ delivery, providing an initial foundation for future optimization of FmocFF hydrogels with an outlook in therapeutic applications. As more small anticancer peptide drugs will emerge (ref. [58]), this approach could be of general interest. A future challenge in the field would be the integration of theoretical and experimental approaches for encapsulation of multiple drugs within peptide hydrogel matrices and their delivery. Moreover, controlled release approaches could be combined with selective targeting [59], always with the appropriate selection of targeting cues to cancer cells. In this way, selective targeting, controlled release, and, eventually, intracellular monitoring of drugs could offer a viable route for cancer chemotherapy.

## Figures and Tables

**Table 1:** Description of the simulated system based on our previous study [39].

System	# Molecules	# Water Molecules (SOL)	# Atoms	L_BOX_ (nm)
BTZ–FmocFF	50–50	48,379	150,387	12.67

## Data Availability

Data obtained in the present research can be made available upon request.

## Supplementary Materials

The following supporting information can be downloaded at: https://www.mdpi.com/article/10.3390/biom15060839/s1, Figure S1: Standard curve graph of BTZ; Figure S2: Gel formation by FmocFF and FmocFF + BTZ; Figure S3: Fmoc-FF background subtraction protocol; Figure S4: Graph of the drug load and encapsulation efficiency of BTZ. Figure S5: UV–Vis spectra of BTZ released over time; Figure S6: FESEM image of the initial BTZ solution and initial FmocFF gel; Figures S7 to S17: Mass spectrometry ion scans of BTZ at time points 1 h to 7 days. Videos S1 and S2: Kinetics of gel formation for FmocFF and FmocFF + BTZ.

## Abbreviations

The following abbreviations are used in this manuscript:

BTZ	Bortezomib
MM	Multiple myeloma
MCL	Mantle cell lymphoma
FmocFF	N-(fluorenylmethyloxycarbonyl)-Phenylalanine-Phenylalanine
HFIP	1,1,1,3,3,3-Hexafluoro-2-propanol
DMSO	Dimethyl sulfoxide
EtOH	Ethanol
AMC	7-amino-4-methylcoumarin
SEM	Scanning Electron Microscopy
FESEM	Field Emission Scanning Electron Microscopy
ESI	Electrospray Ionization
MD	Molecular Dynamics
