# Supplementary material for: FmocFF Peptide Hydrogel Is a Promising Matrix for Encapsulation and Controlled Release of the Anticancer Peptide Drug Bortezomib
Source: Biomolecules. 2025 Jun 8;15(6):839. doi: 10.3390/biom15060839 (PMC12191350; doi:10.3390/biom15060839)
Supplement: Supplementary file 1 [file biomolecules-15-00839-s001.zip › biomolecules-3602415-supplementary.pdf]

biomolecules

Article

FmocFF Peptide Hydrogel Is a Promising Matrix for Encapsulation and Controlled Release of the Anticancer Peptide Drug Bortezomib

Peter Divanach [1,2,†], Antzela Noti [1], Panagiotis Vouvopoulos [1,†], Thanasis Athanasiou [2], Nikos Kountourakis [3], Vagelis Harmandaris [4,5,6], Anastassia N. Rissanou [7,*] and Anna Mitraki [1,2,*]

1 Department of Materials Science and Engineering, University of Crete, Voutes Campus, GR-70013 Heraklion, Greece; petntiv@materials.uoc.gr (P.D.); angelanoti82@gmail.com (A.N.); bio1p502@edu.biology.uoc.gr (P.V.)
2 Institute of Electronic Structure and Laser, Foundation for Research and Technology-Hellas (FORTH), Nikolaou Plastira 100, Vassilika Vouton, GR-70013 Heraklion, Greece; athanasiou@iesl.forth.gr
3 Proteomics Facility, Institute of Molecular Biology & Biotechnology, Foundation for Research and Technology-Hellas (FORTH), Nikolaou Plastira 100, GR-70013 Heraklion, Greece; kountour@imbb.forth.gr
4 Institute of Applied and Computational Mathematics (IACM), Foundation for Research and Technology-Hellas (FORTH), GR-71110 Heraklion, Greece; harman@uoc.gr
5 Department of Mathematics and Applied Mathematics, University of Crete, GR-71409 Heraklion, Greece
6 Computation-Based Science and Technology Research Center, The Cyprus Institute, Nicosia 2121, Cyprus
7 Theoretical & Physical Chemistry Institute, National Hellenic Research Foundation, 48 Vassileos Constantinou Avenue, GR-11635 Athens, Greece
* Correspondence: trissanou@eie.gr (A.N.R.); mitraki@materials.uoc.gr (A.M.)
† These authors contributed equally to this work.

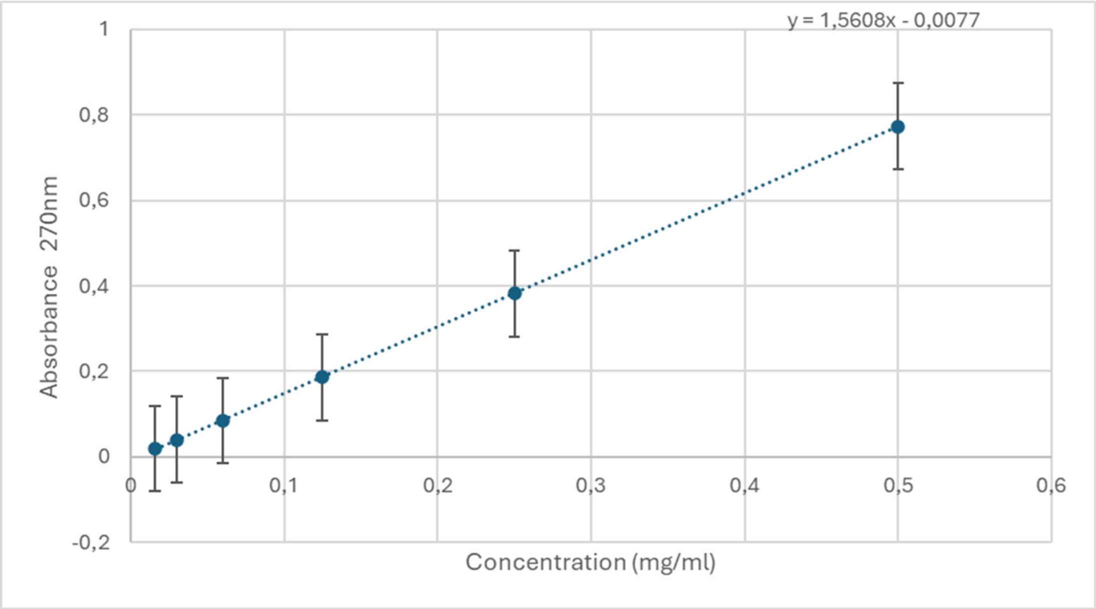

Figure S1: Standard curve graph of BTZ**.** The reference curve was obtained for the pure BTZ solution by measuring the absorbance in replicates at the 270 nm wavelength for the following concentrations: c=0.5, 0.25, 0.125, 0.06, 0.03, and 0.016 mg/ml, as described in the Materials and Methods section.

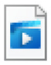

FmocFF.mp4

Supporting Video S1: Mixing FmocFF dissolved in "good solvent" (ethanol) with "bad solvent" (water) and kinetics of structural transition and gel formation.

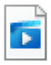

FmocFF+BTZ.mp4

Supporting Video S2: Mixing FmocFF+BTZ dissolved in "good solvent" (ethanol) with "bad solvent" (water) and kinetics of structural transition and gel formation.

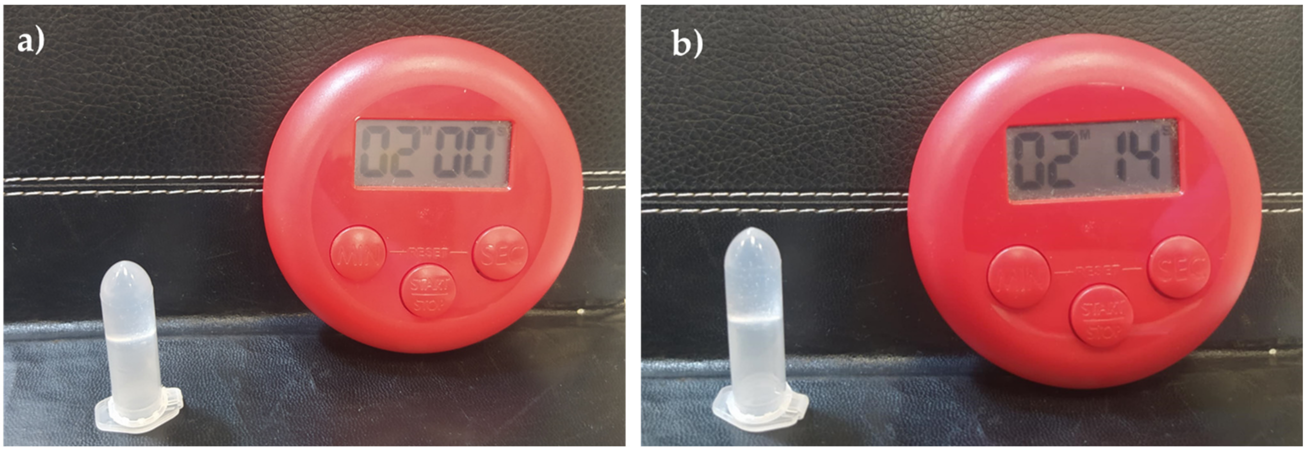

Figure S2: Gel formation by FmocFF and FmocFF+BTZ Inverted Eppendorf tubes showing self-supporting gels formed by FmocFF (a) and FmocFF+BTZ (b).

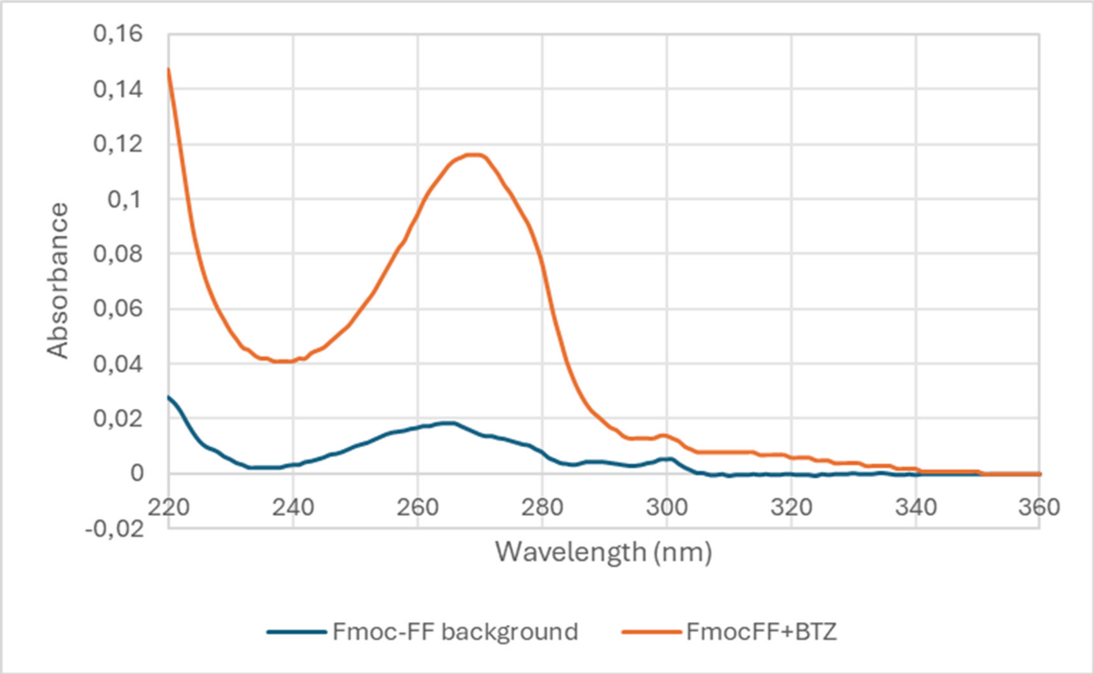

Figure S3: Fmoc-FF background subtraction protocol. Spectrum of BTZ and the spectrum of Fmoc FF in the eluate collected at 48 hours of release. The contribution of Fmoc-FF at 270 nm is subtracted at each time point of release to accurately calculate the quantity of BTZ released.

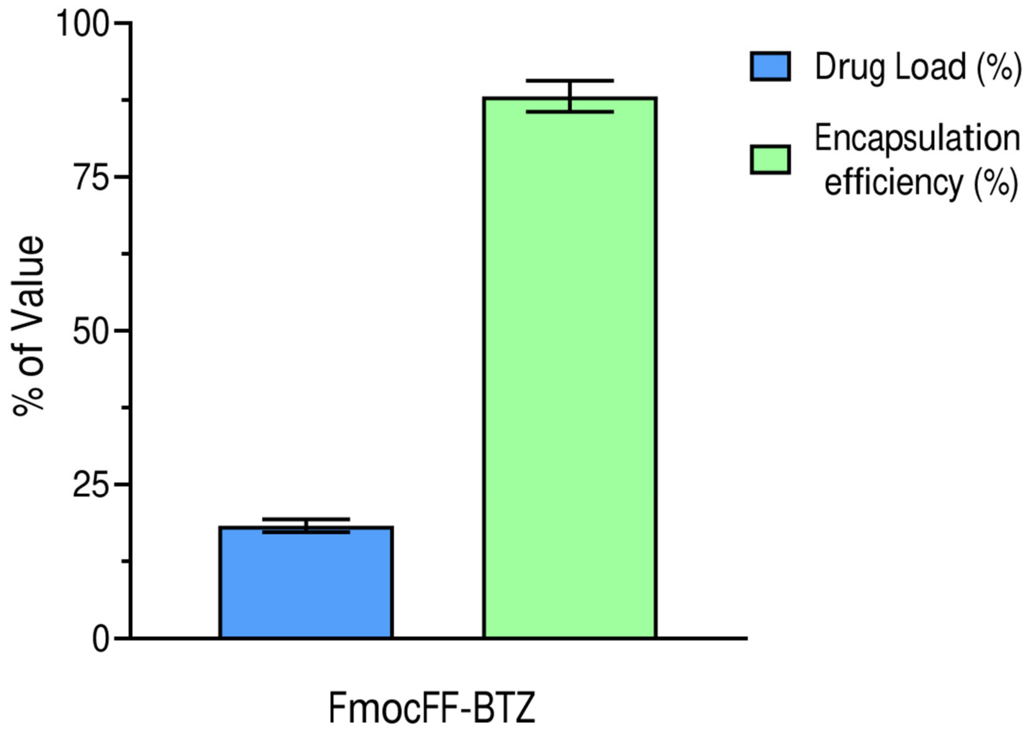

Figure S4: Drug load and encapsulation efficiency of BTZ in FmocFF (mean±SD, n=3).

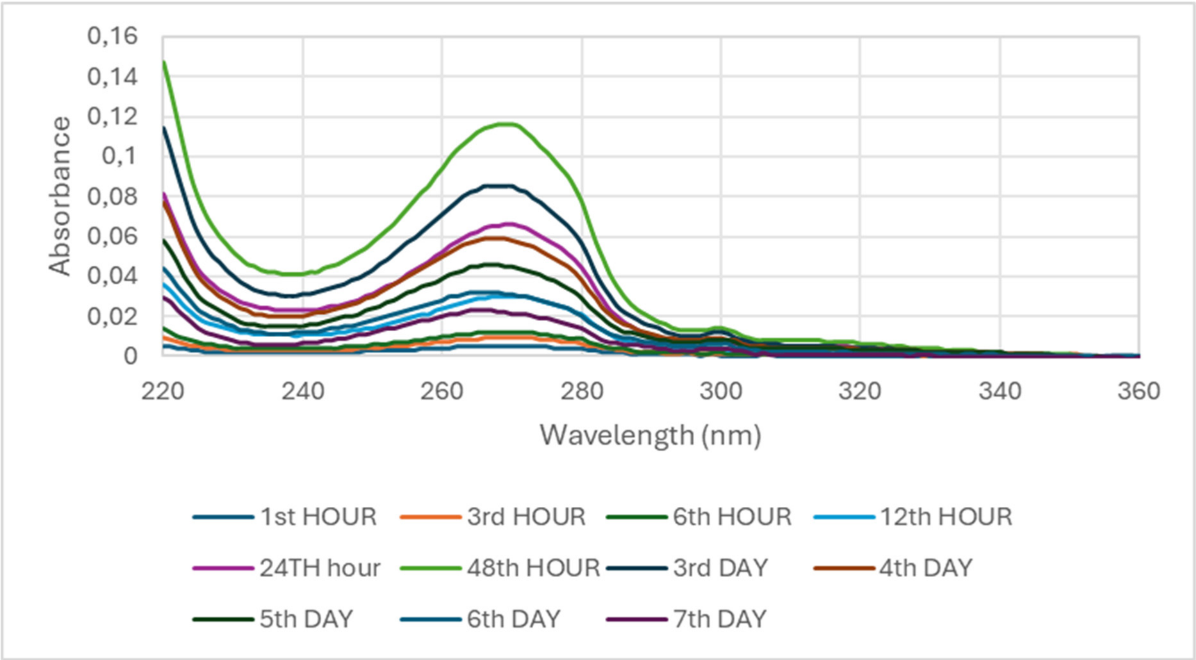

Figure S5: UV–Vis spectra of BTZ released over time (one representative sample out of a total of 13).

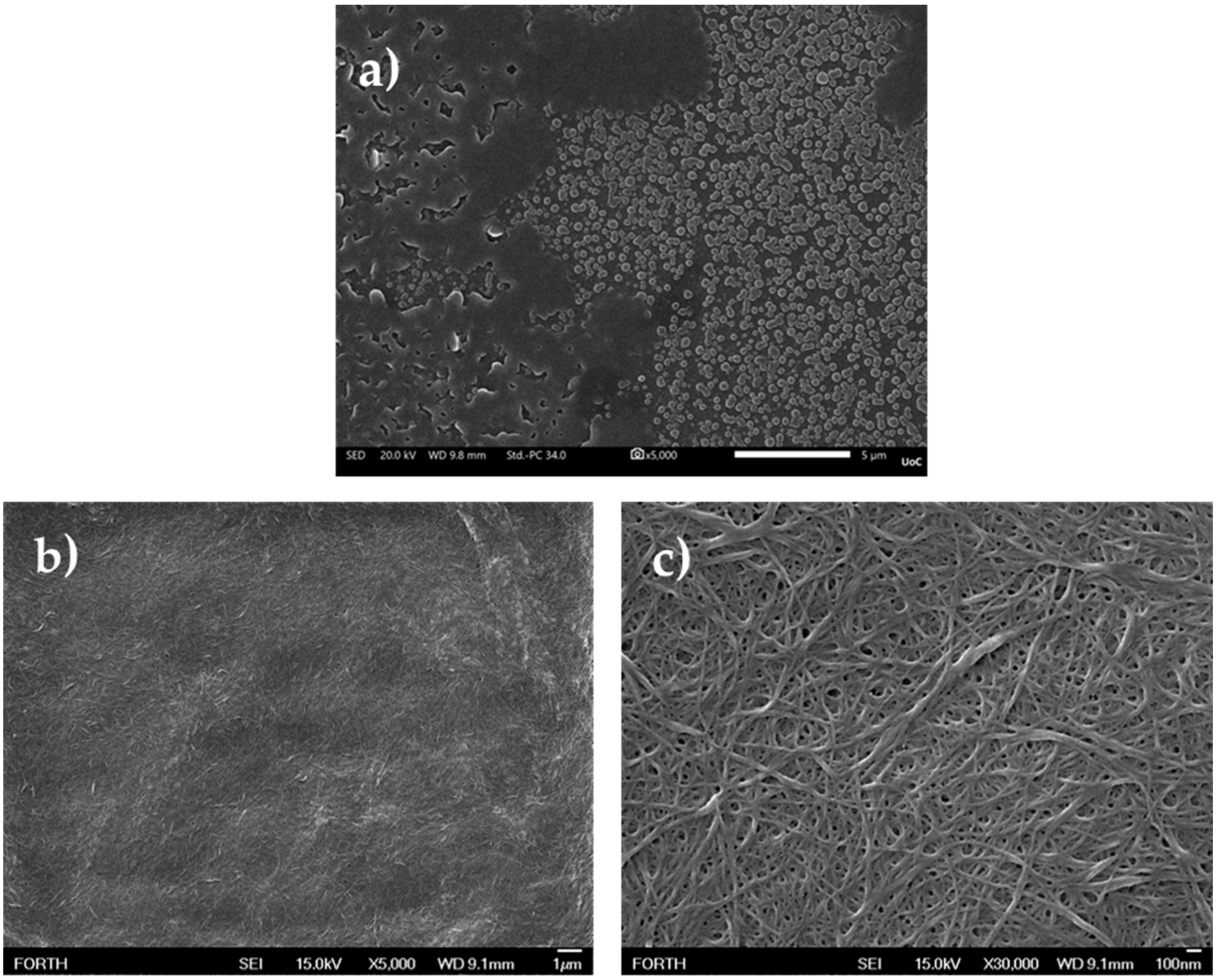

Figure S6: FESEM images of the BTZ and FmocFF. **a)** initial solution of BTZ at 0.5 mg/ml. Scale bar: 5 μm. **b)** Initial, undiluted FmocFF (2mg/ml) gel. scale bar: 1 5 μm. **c)** The same sample at higher magnification in order to better visualize the fibrous network. Scale bar=100 nm.

Mass Spectrometry analysis:

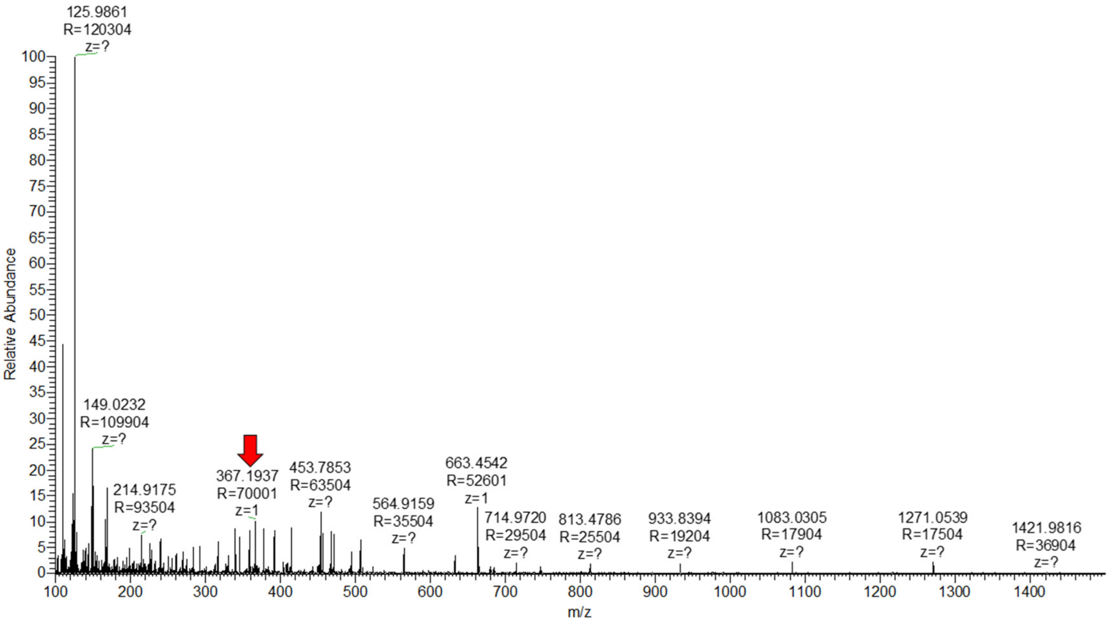

Figure S7: Product ion scan of BTZ (precursor m/z 367.19) obtained in ESI positive ion mode for the 1-hour FmocFF – BTZ release-sample in water at 37 ºC.

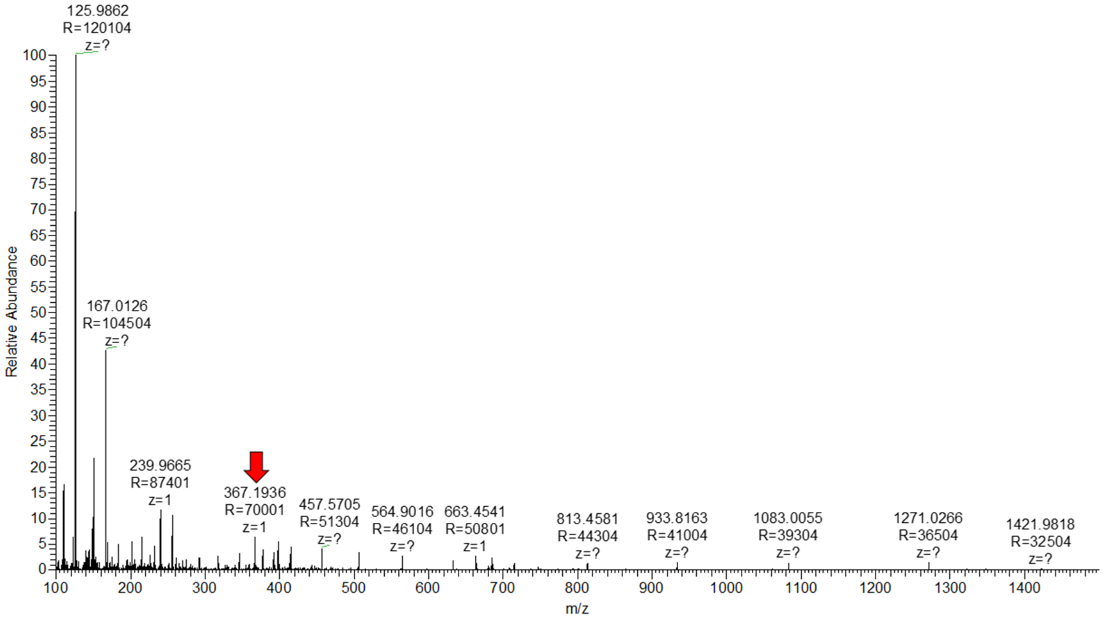

Figure S8: Product ion scan of BTZ (precursor m/z 367.19) obtained in ESI positive ion mode for the 3-hour FmocFF – BTZ release-sample in water at 37 ºC.

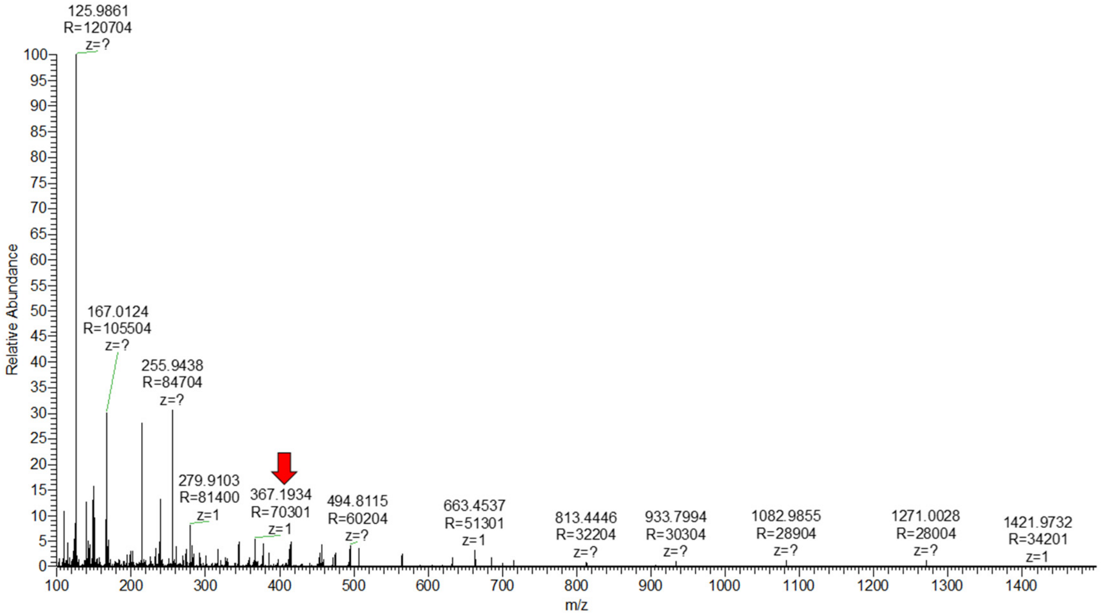

Figure S9: Product ion scan of BTZ (precursor m/z 367.19) obtained in ESI positive ion mode for the 6-hour FmocFF – BTZ release-sample in water at 37 ^{o}C.

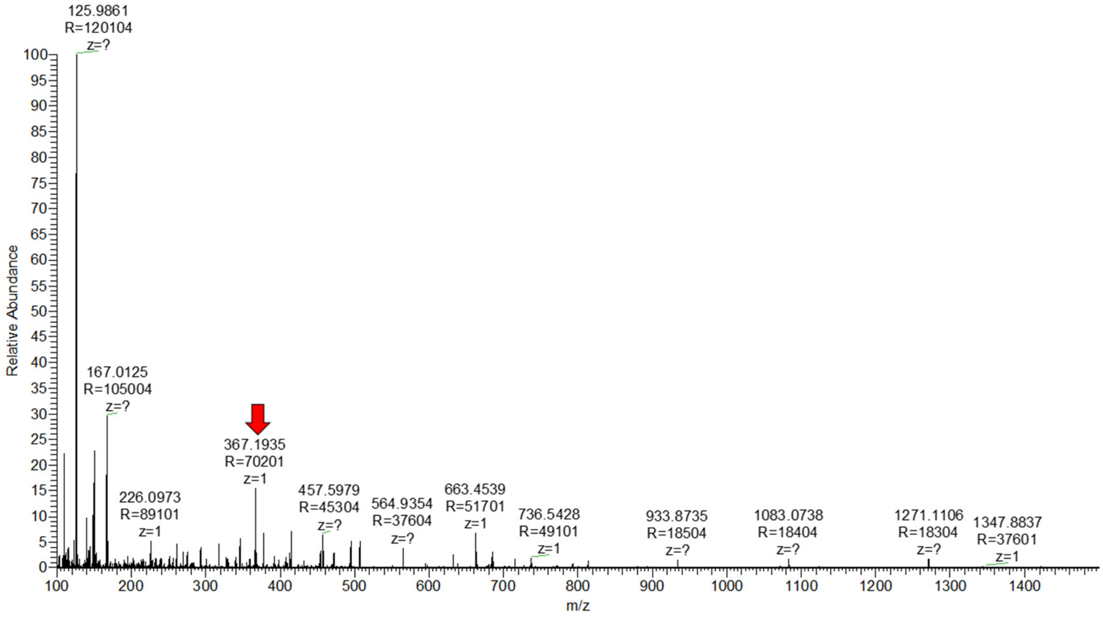

Figure-S10: Product ion scan of BTZ (precursor m/z 367.19) obtained in ESI positive ion mode for the 12-hour FmocFF – BTZ release-sample in water at 37 ^{o}C.

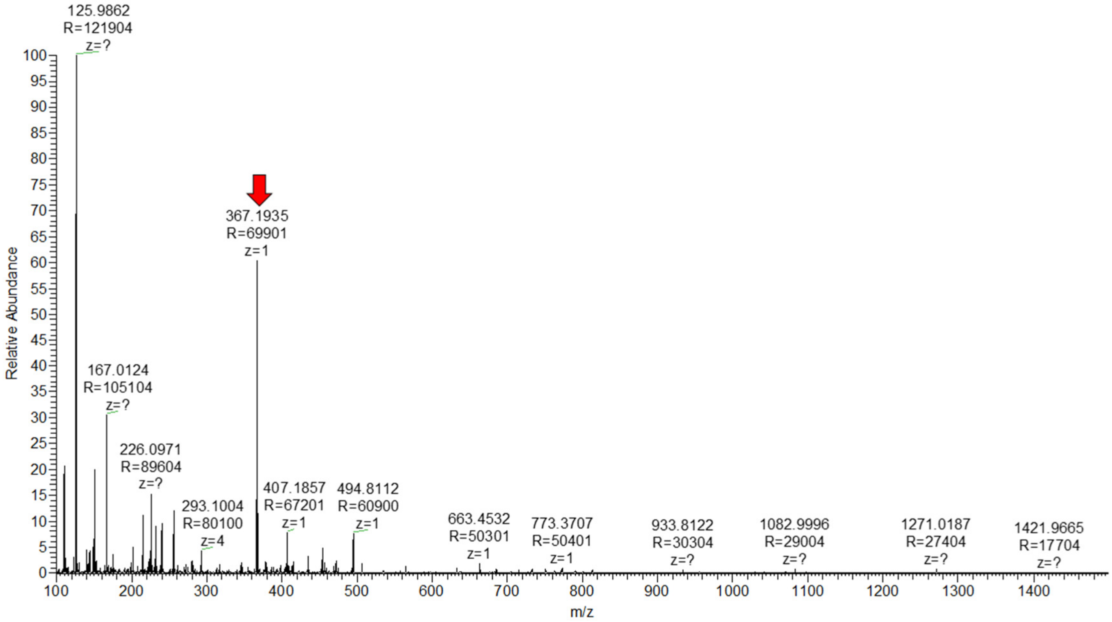

Figure S11: Product ion scan of BTZ (precursor m/z 367.19) obtained in ESI positive ion mode for the 24-hour FmocFF – BTZ release-sample in water at 37 °C.

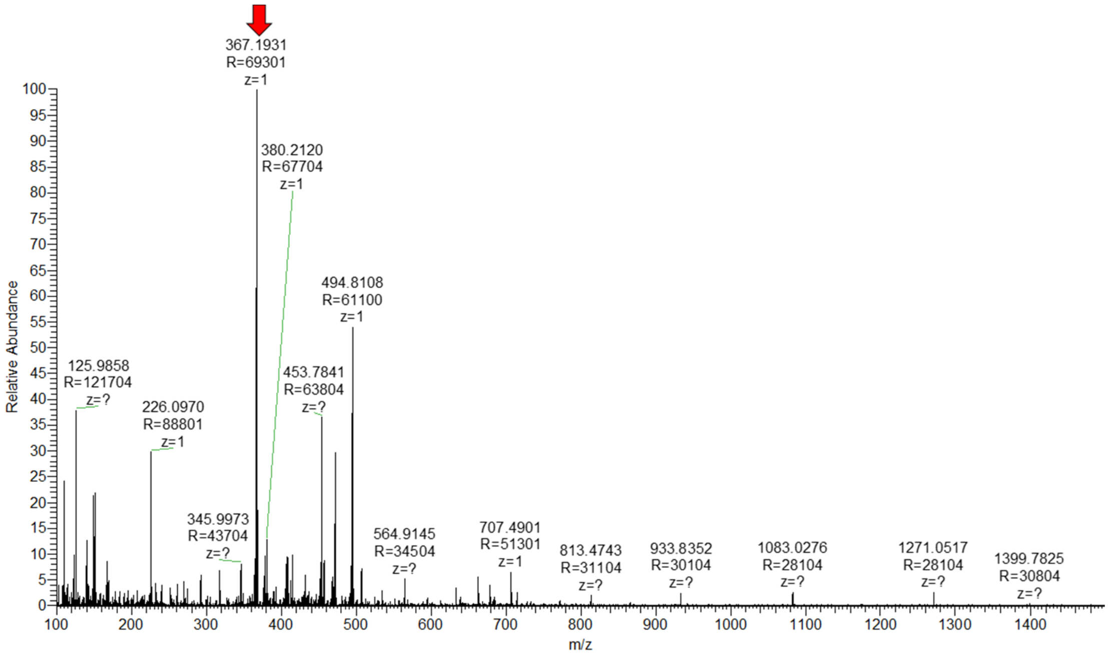

Figure S12: Product ion scan of BTZ (precursor m/z 367.19) obtained in ESI positive ion mode for the 48-hour FmocFF – BTZ release-sample in water at 37 °C.

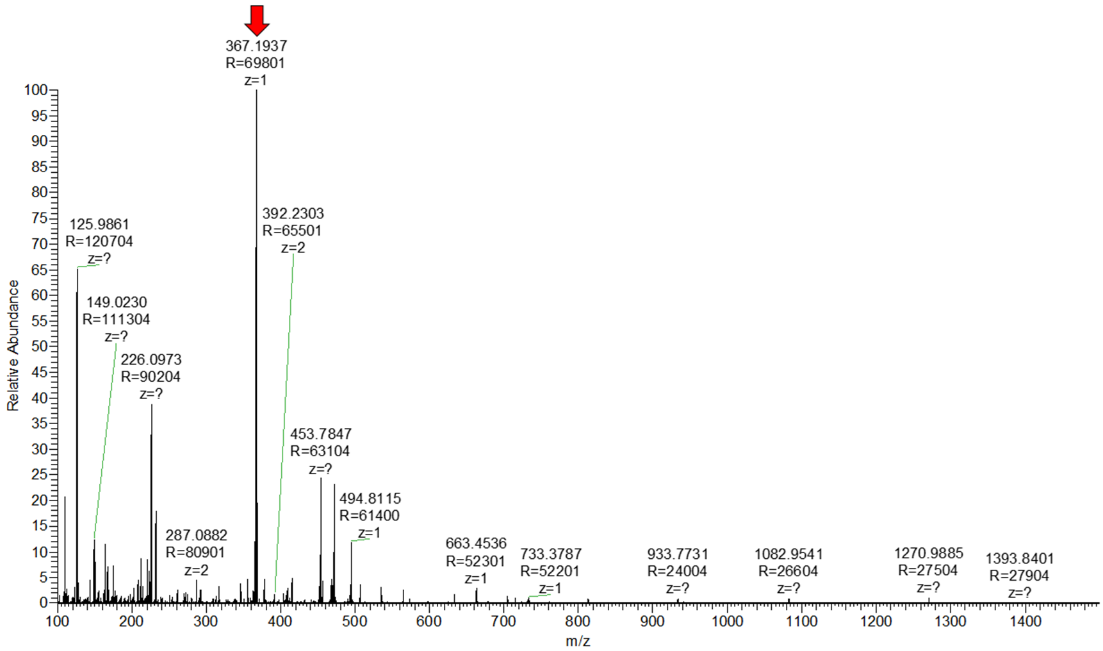

Figure S13: Product ion scan of BTZ (precursor m/z 367.19) obtained in ESI positive ion mode for the 3-day FmocFF – BTZ release-sample in water at 37 ºC.

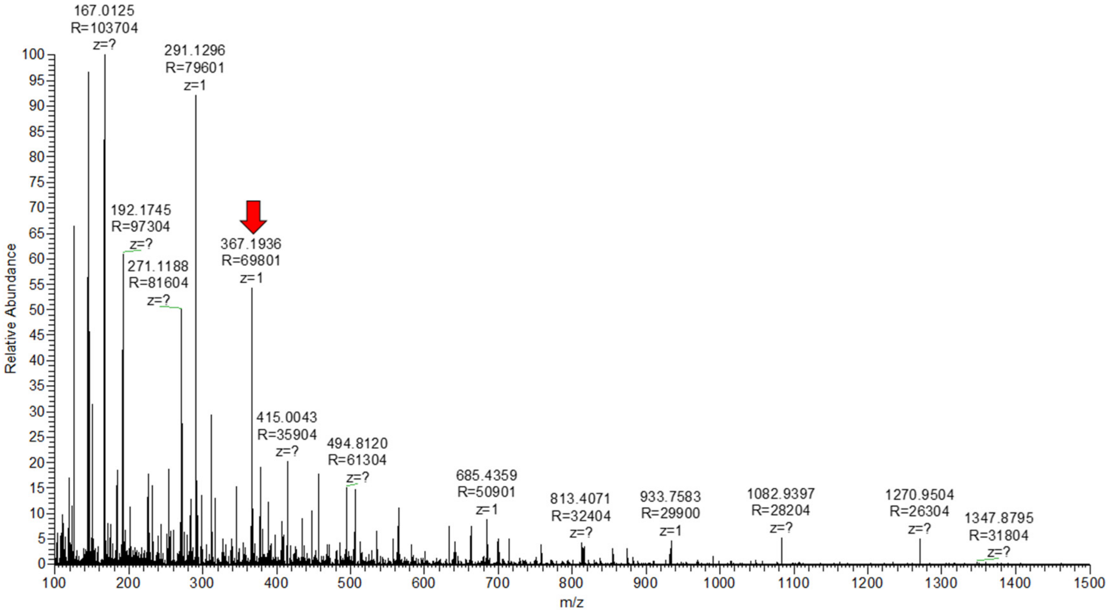

Figure S14: Product ion scan of BTZ (precursor m/z 367.19) obtained in ESI positive ion mode for the 4-day FmocFF – BTZ release-sample in water at 37 ºC.

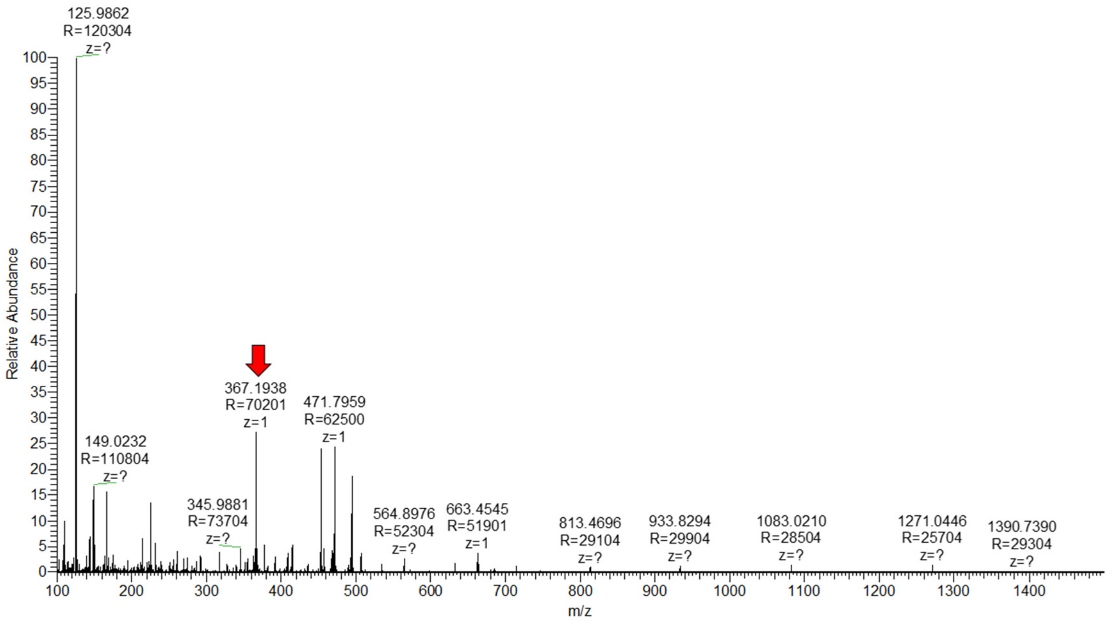

Figure S15: Product ion scan of BTZ (precursor m/z 367.19) obtained in ESI positive ion mode for the 5-day FmocFF – BTZ release-sample in water at 37 °C.

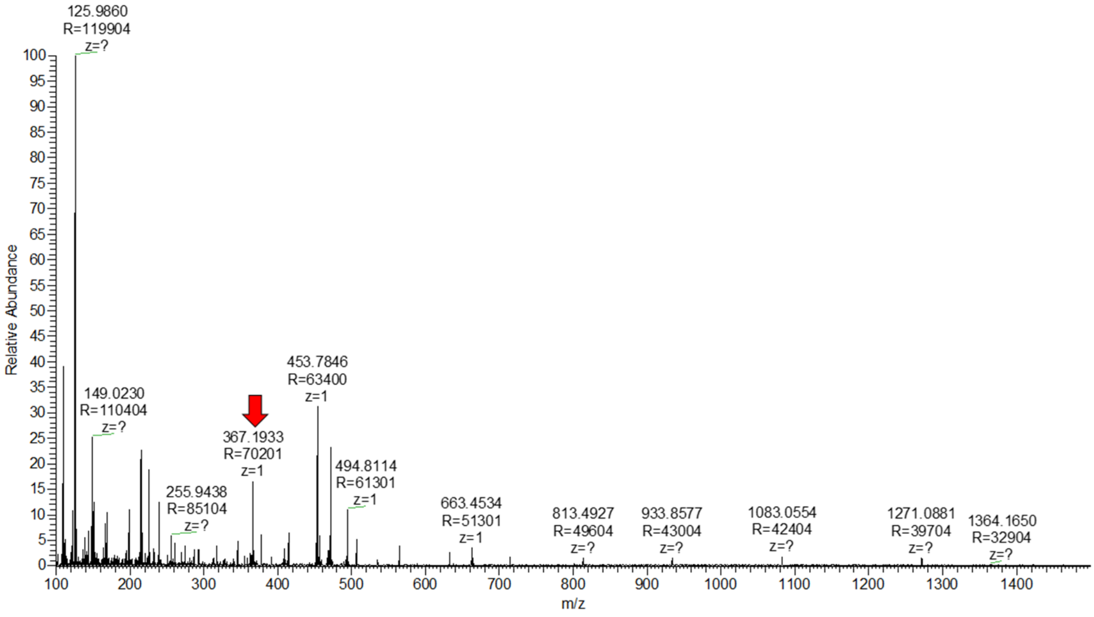

Figure S16: Product ion scan of BTZ (precursor m/z 367.19) obtained in ESI positive ion mode for the 6-day FmocFF – BTZ release-sample in water at 37 °C.

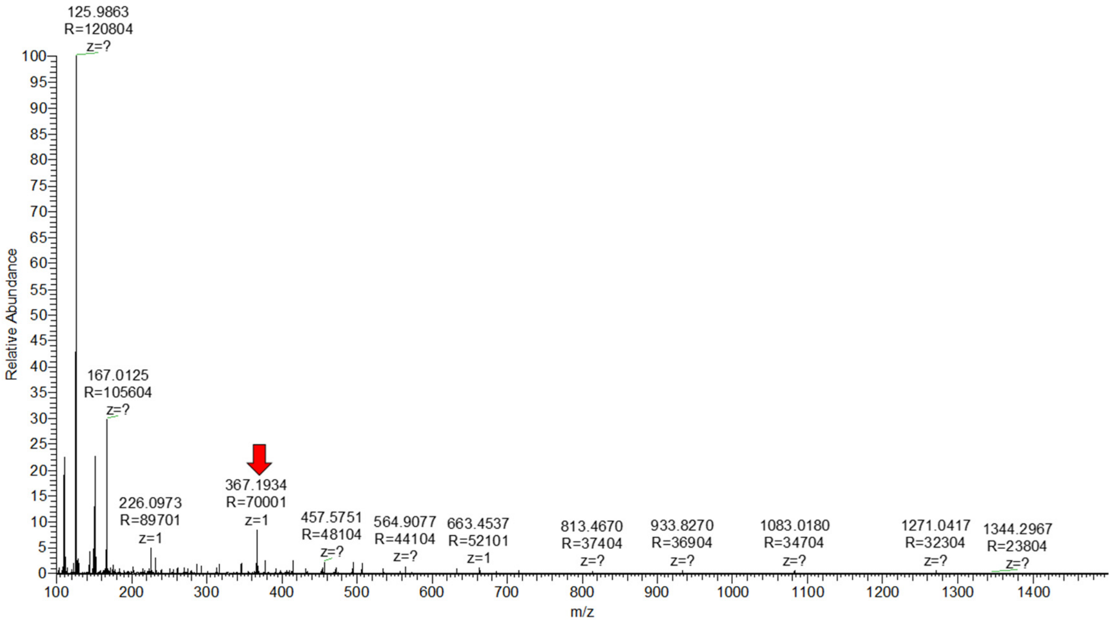

Figure S17: Product ion scan of BTZ (precursor m/z 367.19) obtained in ESI positive ion mode for the 7-day FmocFF – BTZ release-sample in water at 37 °C.

Peaks from FmocFF—BTZ release-samples were detected in positive ion mode with the help of an ESI source and the Orbitrap analyzer. The relative intensities of the peaks were much higher for the 48-hour sample and the 3-day sample, followed by the 24-hour samples and the 4- and 5-day samples, respectively. The 12-hour and 6-day samples displayed similar BTZ relative intensities that were lower than the aforementioned samples but higher than the 1-week sample. Finally, the lowest BTZ relative intensities were observed for the early 1-hour, 3-hour, and 6-hour samples.
